# Large Improvement of the Mechanical Strength of Carbon Nanotube Films by Joule Heating Dominated Post Treatments

**DOI:** 10.3390/ma19132917

**Published:** 2026-07-07

**Authors:** Zujia Hu, Yifan Feng, Heng Zhang, Kangfei Liu, Xinran Cheng, Yunxiao Du, Jiannong Wang

**Affiliations:** 1 School of Materials Science and Engineering, East China University of Science and Technology, 130 Meilong Road, Shanghai 200237, China; y30230843@mail.ecust.edu.cn (Z.H.); y30230806@mail.ecust.edu.cn (Y.F.); y10220085@mail.ecust.edu.cn (H.Z.); y30230783@mail.ecust.edu.cn (X.C.); y30240867@mail.ecust.edu.cn (Y.D.); 2 School of Mechanical and Power Engineering, East China University of Science and Technology, 130 Meilong Road, Shanghai 200237, China; y15230004@mail.ecust.edu.cn

**Keywords:** CNT assemblies, resistance heating, defect passivation, interfacial enhancement, tensile performance

## Abstract

**Highlights:**

- A synergistic three-step post-treatment (acid washing + pre-stretching + Joule heating) is developed to substantially enhance the mechanical properties of FCCVD-grown CNT films.
- Joule heating purification mechanism is clarified: for high-iron films (>30 wt.%), iron melts and forms Fe/Fe_3_C@C core–shell particles; for low-iron films, iron is removed via atomic diffusion and evaporation.
- Joule heating converts weak van der Waals contacts into continuous carbon networks at tube junctions, repairing sp^2^ defects (I_G_/I_D_ from 2.33 to 3.12) and locking the pre-stretched alignment.
- The final CNT film achieves a specific strength of 7.04 N/tex and a true tensile strength of 8.05 GPa after rolling, surpassing most reported carbon fibers and CNT fibers.

**Abstract:**

Carbon nanotube (CNT) films prepared via floating catalyst chemical vapor deposition generally suffer from residual iron impurities, structural defects, and weak inter-tube interfaces, which severely limit their mechanical performance. Here, we propose a post-treatment approach, which is dominated by Joule heating, to substantially improve the mechanical properties of CNT films. Acid washing after Joule heating effectively removes iron catalyst and amorphous carbon, increasing the specific strength from 0.64 N/tex to 2.96 N/tex. Pre-stretching induces alignment of the CNTs along the stretching direction, further raising the specific strength to 5.57 N/tex. Subsequent Joule heating not only raises graphitization degree and repairs lattice defects but also transforms the weak van der Waals contacts between tubes into continuous carbon networks, leading to network densification and locking of the aligned structure. The final specific strength reaches 7.04 N/tex and true tensile strength 8.05 GPa, surpassing previous representative carbon materials. The purification mechanism of Joule heating depends on the initial iron content of the film: for high-iron films, iron melts, migrates and forms Fe/Fe_3_C@C core–shell particles, which can be converted into hollow carbon shells via acid etching; for low-iron films, iron is removed via atomic diffusion and evaporation. This work provides a fast, controllable and synergistic technical route for the preparation of high-performance CNT macrostructures.

## 1. Introduction

Carbon nanotubes possess excellent mechanical, electrical and thermal properties, and the theoretical tensile strength of an individual CNT can exceed 100 GPa [1,2]. However, when assembled into macroscopic films or fibers, the measured mechanical properties are usually about two orders of magnitude lower than the theoretical value [3]. The main reasons for this sharp discrepancy include the presence of residual metal catalyst particles and amorphous carbon from the synthesis process, structural defects in the tube walls (such as vacancies, dangling bonds and topological defects), and weak inter-tube interactions dominated by van der Waals forces. These problems severely limit the load transfer efficiency and structural integrity of CNT macrostructures.

To address these issues, various post-treatment strategies have been developed. Acid washing can remove catalyst impurities, but it tends to damage the CNT network and cannot repair tube-wall defects [4,5]. Mechanical stretching can improve preferential alignment and packing density of CNTs, but the inter-tube interfaces remain physical contacts that are prone to sliding [6,7]. Conventional thermal annealing can reduce defects and improve the graphitization degree, but suffers from long processing times, high energy consumption, and difficulty in selectively acting on the contact regions [3].

In recent years, Joule heating has emerged as a promising technique for processing materials [8,9,10]. By passing an electric current directly through a conductive carbon material, intense resistive heating can be generated within seconds, raising the temperature above 2000 K. This ultrafast, energy-efficient and locally applicable heating method has been successfully applied to carbon nanofibers and graphene oxide papers for achieving purification, defect repair and even covalent bonding between adjacent fibers [3,11,12]. Nevertheless, systematic studies on CNT materials are still lacking, regarding impurity control, structural evolution, and mechanical reinforcement [13,14,15,16]. In particular, the mechanisms of purification and structural transformation and their synergistic effects on mechanical properties under Joule heating remain unclear [17,18,19,20].

In this work, we demonstrate an approach to significantly enhancing the mechanical strength of CNT films by Joule heating. That is, after proper purification and CNT alignment, a controlled Joule heating treatment is applied to repair tube-wall defects, enhance inter-tube interfaces, densify the network and lock the aligned structure. In doing so, the purification effect of Joule heating on high-iron and low-iron films is compared, and the microstructural evolution at each step is systematically characterized via electron microscopy, Raman spectroscopy and mechanical testing. The synergistic effects are discussed, and a multi-scale reinforcement mechanism is proposed. Using this method, the specific strength of the CNT film is increased from 0.64 N/tex to 7.04 N/tex with a final tensile strength of 8.05 GPa, demonstrating a fast and controllable route to high-performance CNT macrostructures.

## 2. Materials and Methods

### 2.1. Preparation of CNT Films

**Figure 1:**
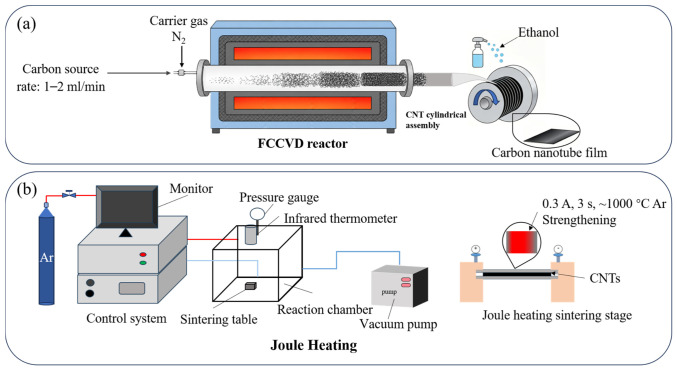
Schematic illustration of the fabrication and post-treatment processes of carbon nanotube (CNT) films. (**a**) FCCVD fabrication; (**b**) Joule heating treatment.

CNT films were synthesized using a floating catalyst chemical vapor deposition (FCCVD) method (Figure 1a). Ethanol was used as the carbon source, thiophene as the promoter, and ferrocene as the catalyst precursor. The precursor solution was prepared by dissolving ferrocene in ethanol with the addition of thiophene, followed by ultrasonic agitation to ensure complete mixing. The solution was continuously injected into a horizontal tube furnace, with nitrogen as the carrier gas. The furnace temperature was maintained at approximately 1150 °C. In the high-temperature zone, the carbon source decomposed and CNTs grew on the floating catalyst particles. The CNT assembly was then carried by the gas flow out of the furnace and collected on a rotating drum wrapped with an aluminum foil. During the collection, ethanol was sprayed onto the CNT network to densify the film via surface tension. After natural drying, a self-supporting CNT film was obtained.

Two types of CNT films with different iron contents were synthesized by varying the catalyst concentration and growth parameters. For the low-iron-content films (C_Fe_ < 30 wt.%), the ferrocene concentration was fixed at 0.2 wt.%, thiophene at 0.05 wt.%, the precursor injection rate at 2 mL/min, and the N_2_ flow rate at 600 mL/min. These low-iron films were used for the subsequent acid washing, pre-stretching, and Joule heating treatments, as well as for direct Joule heating experiments. For the high-iron-content films (C_Fe_ > 30 wt.%), the ferrocene concentration was increased to 1.0 wt.%, thiophene to 0.3 wt.%, the injection rate to 4 mL/min, and the N_2_ flow rate to 1000 mL/min. These high-iron films were used exclusively for direct Joule heating treatment to investigate the iron-removal mechanism.

### 2.2. Post-Treatments

Acid washing: The as-prepared CNT films were cut into strips of approximately 2 mm × 10 cm. Chlorosulfonic acid (CSA) was diluted with dichloromethane to volume fractions of 5 vol.%, 10 vol.%, 20 vol.%, 30 vol.% and 50 vol.%. The CNT strips were immersed in the CSA solution for 1 min, then thoroughly rinsed with ethanol, and finally dried in an oven at 120 °C for 4 h. The optimal condition (treated with 30 vol.% CSA) was selected for subsequent pre-stretching and Joule heating experiments.

Pre-stretching: After acid washing with 30 vol.% CSA (the optimal condition determined from mechanical tests), the CNT strips were pre-stretched. One end of the strip was fixed at one end, and a glass weight of 50 g, 100 g or 150 g was hung on the other end. The stretching lasted for 30 min (a separate set of samples was stretched at 100 g for 3 h for comparison). After stretching, the strips were dried at 120 °C for 4 h to fix the aligned structure. Pre-stretching treatment applied with a 100 g weight for 30 min was identified as the optimal condition and used for the subsequent Joule heating treatments.

Joule heating: After acid washing and pre-stretching under the optimal conditions described above, the CNT strips were subjected to Joule heating in an argon atmosphere using a custom-built apparatus (HTS-5030D, Shenzhen Zhongke Jingyan Technology Co., Ltd., Shenzhen, China) as shown in Figure 1b. The strips were clamped between two electrodes, and a direct current of 0.1 A, 0.3 A or 0.5 A was applied for 3 s, corresponding to temperatures of approximately 800 °C, 1000 °C and 1800 °C as measured using an infrared pyrometer (model RAYR3I1ML3; calibrated by the manufacturer with a factory calibration certificate; emissivity setting ε = 0.90). The pyrometer was focused on the central area of the CNT strip, and the temperature uncertainty is estimated to be approximately ±50 °C based on the calibration and measurement conditions. The heating was performed in a sealed chamber with continuous argon flow to prevent oxidation. It should be noted that these samples are distinct from J-CNT1 and J-CNT2, which were obtained via directly Joule heating the as-prepared low-iron-content films at 32 V and 50 A (>1400 °C) without acid washing or pre-stretching (Section 3.1).

### 2.3. Characterization

Morphology and structure: The surface morphology and cross-section of the CNT films were observed using field-emission scanning electron microscopy (SEM, GeminiSEM500, Munich, Germany, Zeiss) at an accelerating voltage of 5 kV. Transmission electron microscopy (TEM, JEM-2100, JEOL, Tokyo, Japan) was performed at 200 kV to examine the tube wall structure and defects. For TEM observation, CNTs from the high-temperature reactor were directly deposited onto a copper micro grid.

Composition and phase analysis: X-ray diffraction (XRD) patterns were collected using an X-ray photoelectron spectrometer (ESCALAB250Xi, Thermo Fisher, Waltham, MA, USA) with monochromated Al Kα radiation (1486.6 eV). Energy-dispersive X-ray spectroscopy (EDS) was used for elemental analysis. Thermogravimetric analysis (TGA, Q1000, TA Instruments, New Castle, DE, USA) was carried out in oxygen atmosphere (flow rate 50 mL/min) from room temperature to 900 °C at a heating rate of 10 °C/min to determine the iron content of the films. The functional groups and near-surface elemental compositions of the VACNTs were investigated via X-ray photoelectron spectroscopy (XPS, K-Alpha, Thermo Scientific, Waltham, MA, USA).

Raman spectroscopy: Polarized Raman spectra were acquired using a confocal Raman microscope (LABRAM HR, Horiba, Kyoto, Japan) with a 532 nm excitation laser. The orientation parameter was estimated from the G-peak intensity ratio measured with the laser polarization parallel and perpendicular to the film axis.

Mechanical testing: The linear density of the CNT strips was measured using a vibroscopic method (fiber fineness tester) with a gauge length of 20 mm. Tensile tests were performed on a single-fiber tensile testing machine (XS (08)X-15, range 15 N, resolution 0.01 cN) with a gauge length of 10 mm at a stretching rate of 20 mm/min. The cross-sectional area of the strips was determined from SEM images of their cross-sections. At least three specimens were tested for each set of films, and the average values are reported.

## 3. Results and Discussion

### 3.1. Purification of High-Iron and Low-Iron Content CNT Films by Direct Joule Heating

**Figure 2:**
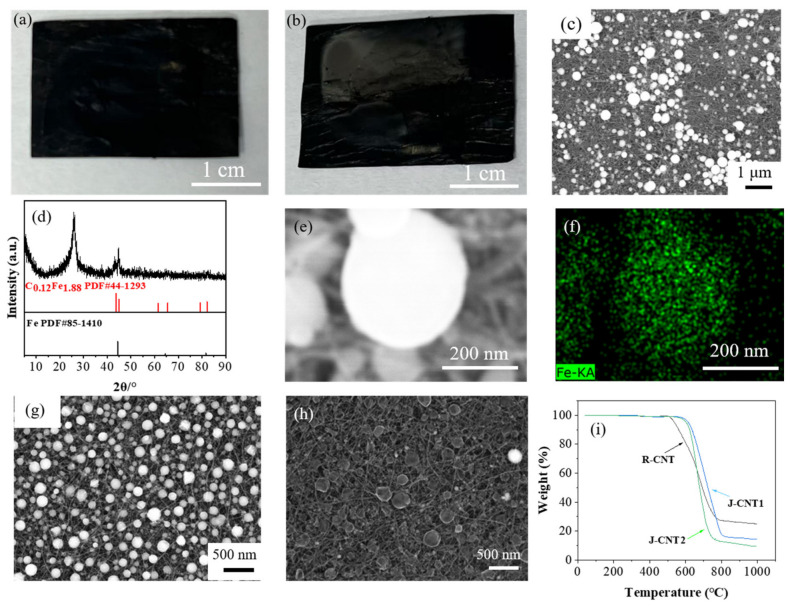
Purification of CNT films by direct Joule heating. (**a**–**h**) Data from the high-iron content film treated at 32 V, 50 A, >1400 °C; (**a**,**b**) Optical photographs before and after Joule heating. (**c**) SEM image of spherical particles. (**d**) XRD pattern of the particles. (**e**) High-magnification SEM image showing core–shell structure. (**f**) EDS spectrum. (**g**,**h**) SEM image before and after acid washing. (**i**) TGA curves of CNT films before (R-CNT) and after Joule heating (J-CNT1, J-CNT2).

Joule heating, as a rapid and short-duration thermal treatment, can effectively purify and strengthen CNT films. This study aims to utilize this technique to produce CNT films with both high strength and high purity. To evaluate the purification capability of Joule heating on CNT films, the large-sized films (2 cm × 3 cm) shown in Figure 2a were used for the Joule heating experiments, rather than the small-sized films employed for mechanical property measurements in the subsequent sections. Owing to the significantly larger dimensions, the current was increased to 50 A to reach temperatures sufficient for effective purification, in contrast to the 0.1–0.5 A range used for the small-sized samples. High-iron-content CNT films (pristine Fe content ≈ 35 wt.%, determined via TGA) were treated via direct Joule heating (32 V, 50 A, 5 s, >1400 °C, Ar atmosphere). After treatment, the film surface turned from uniform black to a pattern densely covered with white particles (Figure 2a,b). SEM imaging revealed that these particles were spherical, with diameters on the micrometer scale, and were scattered across the CNT network or lodged in the inter-tube spaces (Figure 2c).

X-ray diffraction (XRD) analysis of the white particles (Figure 2d) showed characteristic peaks corresponding to Fe_3_C and metallic Fe, while no iron oxide peaks were detected, consistent with the inert argon atmosphere. Energy-dispersive X-ray spectroscopy (EDS) and SEM confirmed that the particles were rich in iron (Figure 2e,f). These observations indicate that the iron catalyst originally embedded in the film melted at temperatures close to the melting point of iron (1538 °C), migrated to the surface, and solidified into spherical droplets under the driving force of surface tension.

EDS (Figure 2f) further revealed that each spherical particle was encapsulated by a thin, continuous carbon shell, forming a core–shell structure (Fe/Fe_3_C@C). The formation of this graphitic shell is attributed to the catalytic graphitization of amorphous carbon on the iron surface at high temperature. After acid washing (4 mol/L H_2_SO_4_, 4 h) to remove the iron core, the carbon shells remained intact, yielding hollow carbon spheres that still adhered to the CNT network (Figure 2h). This demonstrates that the core–shell particles are selectively etchable, while the outer carbon layer is chemically robust due to its high graphitization degree. Importantly, the acid washing did not visibly damage the underlying CNT network, as confirmed by the retained structural integrity.

To verify whether the purification mechanism of Joule heating is correlated with iron content, Joule heating experiments were also conducted on low-iron-content CNT films under identical conditions. For low-iron-content films (pristine Fe content ≈17.4 wt.%), two parallel Joule heating treatments were conducted on the samples (32 V, 50 A, 5 s, >1400 °C, Ar atmosphere), designated as J-CNT1 and J-CNT2, while the pristine sample was labeled as RCNT. The same Joule heating treatment did not produce any observable particles on the surface. TGA (Figure 2i) showed that the iron content decreased from 17.39 wt.% (pristine) to 10.13 wt.% and 6.62 wt.% in two parallel Joule-heated samples (J-CNT1 and J-CNT2). This indicates iron was removed via atomic diffusion and evaporation rather than migration and agglomeration. Therefore, the purification mechanism of Joule heating depends on the initial iron content: for high-iron films, melting and surface migration dominate; for low-iron films, atomic-scale volatilization is the primary pathway.

However, the high temperature (1400 °C) required for Joule heating purification inevitably caused a marked and irreversible decrease in mechanical properties. In view of this, we abandoned the high-temperature purification route and instead adopted Joule heating under relatively mild conditions solely for mechanical property enhancement. Meanwhile, purification was accomplished by acid washing with 30 vol.% CSA, which effectively removes iron impurities without severely damaging the CNT network.

### 3.2. Mechanical Property Optimization via Acid Washing and Pre-Stretching

**Figure 3:**
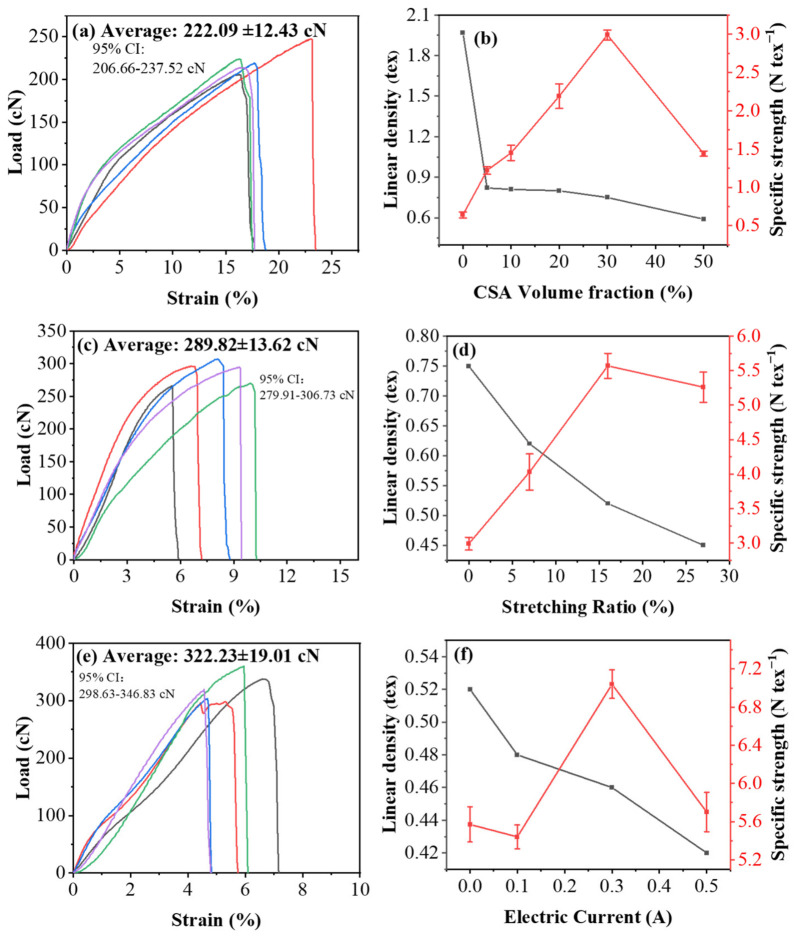
Mechanical properties (Load vs. strain tensile curves and variations of linear density and specific strength with treatment condition) of CNT strips after different post-treatments. In each panel, the five curves represent replicate tests conducted under the same conditions. The error bar indicates the mean ± standard deviation of the specific strength. (**a**,**b**) After acid washing; (**c**,**d**) After pre-stretching; (**e**,**f**) After Joule heating.

To achieve optimal enhancement of mechanical properties, prior to Joule heating, the CNT strips were subjected to acid washing and pre-stretching to remove impurities like amorphous carbon and induce CNT alignment, respectively. Figure S1 shows the tensile load-strain curves of CNT strips after acid washing with different CSA volume fractions (5 vol.%, 10 vol.%, 20 vol.%, 30 vol.% and 50 vol.%) in dichloromethane for 1 min. With increasing CSA concentration, both the tensile load and the strain at break initially increased and then decreased. The optimal performance was observed at 30 vol.% CSA, where the tensile load reached 222.09 cN and the elongation was 18.24% (Figure 3a). The corresponding specific strength (tensile load divided by linear density) increased from 0.64 N/tex for the pristine film to 2.96 N/tex after acid washing (Figure 3b). The linear density decreased from 1.97 tex (pristine) to 0.75 tex, indicating effective removal of partial iron catalyst and amorphous carbon. When the CSA concentration exceeded 30 vol.% (e.g., 50 vol.%), the specific strength dropped to 1.44 N/tex, suggesting that the acid with a high concentration damaged the CNT network.

After acid washing with 30 vol.% CSA, the CNT strips were pre-stretched under different loads (50 g, 100 g and 150 g) for 30 min. A separate set of samples was stretched at 100 g for 3 h for comparison. Figure S2 presents the tensile load-strain curves of the pre-stretched strips. The best mechanical performance was obtained at a load of 100 g for 30 min, where the specific strength further increased to 5.57 N/tex and the tensile load reached 289.82 cN (Figure 3c,d). The linear density decreased to 0.52 tex, reflecting densification due to CNT alignment and stretching. The elongation at break decreased from 18.24% (after acid washing) to 7.29% (after pre-stretching), indicating a transition from ductile to more brittle behaviour, consistent with reduced inter-tube sliding after alignment. Extending the pre-stretching time to 3 h at 100 g did not further improve the specific strength (5.31 N/tex vs. 5.57 N/tex), suggesting that 30 min is sufficient to achieve near-complete orientation. A load of 150 g caused a decline in both specific strength (5.26 N/tex) and tensile load (236.50 cN), indicating that overload led to structural damage such as tube breakage or interfacial debonding.

**Figure 4:**
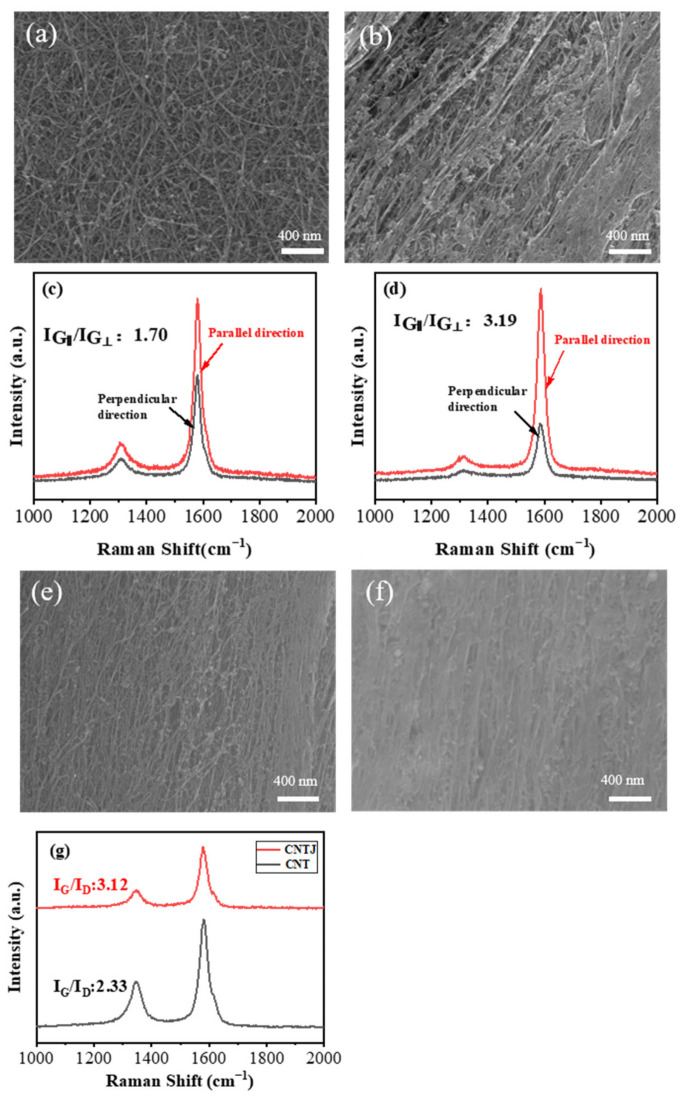
Structural evolution induced by pre-stretching and Joule heating. (**a**,**b**) SEM images before and after pre-stretching. (**c**,**d**) Polarized Raman spectra before and after pre-stretching. (**e**,**f**) SEM images before and after Joule heating. (**g**) Raman spectra before (R-CNT) and after 0.3 A Joule heating (J-0.3 A).

The structural changes induced by pre-stretching were examined via SEM and polarized Raman spectroscopy. As shown in Figure 4, the originally disordered CNT (Figure 4a) network became highly aligned along the stretching direction after pre-stretching (Figure 4b). The polarized Raman spectra (Figure 4c,d) further confirmed this alignment: after pre-stretching, the G-peak intensity showed a strong dependence on the polarization angle, in contrast to the weak angular dependence in the pristine sample. The orientation parameter, defined as the ratio of the G-peak intensity at parallel and perpendicular polarizations, increased significantly, indicating that the CNTs were preferentially oriented. Thus, acid washing with 30 vol.% CSA effectively removed impurities without severely damaging the CNT network, and pre-stretching with a 100 g load produced well-aligned, densified CNT strips with a specific strength of 5.57 N/tex, providing a suitable starting structure for the subsequent Joule heating.

### 3.3. Further Strengthening by Joule Heating

After acid washing (30 vol.% CSA) and pre-stretching (100 g, 30 min), the CNT strips were subjected to Joule heating under argon atmosphere at different currents (0.1 A, 0.3 A and 0.5 A) for a fixed duration of 3 s, corresponding to estimated temperatures of 800 °C, 1000 °C and ≈1800 °C, respectively. Figure S3 shows the tensile load-strain curves of the CNT strips after Joule heating. The best mechanical performance was achieved at 0.3 A (≈1000 °C), where the specific strength reached 7.04 N/tex and the average tensile load increased to 322.23 cN (Figure 3e,f). The linear density further decreased to 0.42 tex, indicating additional densification and purification. At a lower current of 0.1 A (≈800 °C), the temperature was insufficient to activate significant structural changes, resulting in a moderate specific strength of 5.44 N/tex. At a higher current of 0.5 A (≈1800 °C), the specific strength dropped to 5.70 N/tex, suggesting that excessive thermal input causes structural degradation, such as carbon vaporization or the generation of new defects.

**Figure 5:**
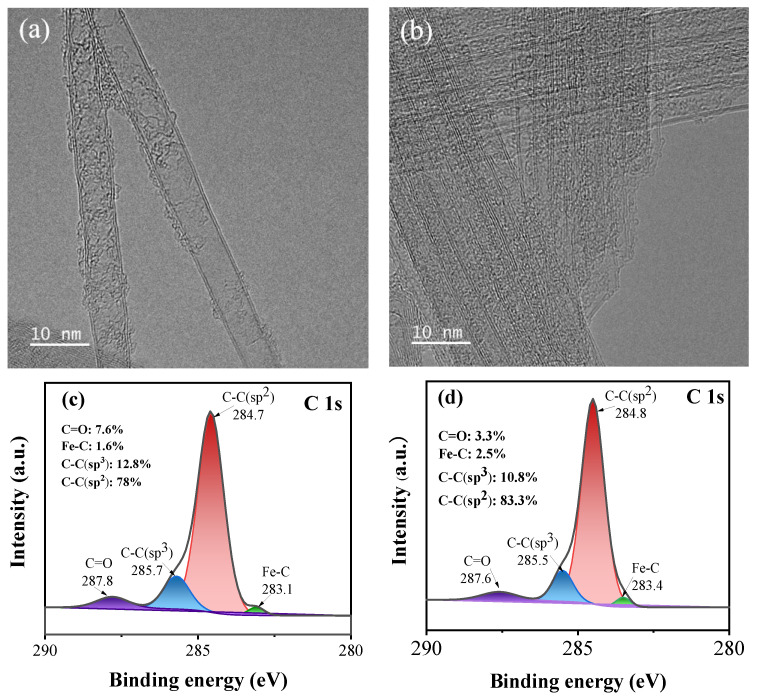
HRTEM images of CNTs (**a**) before Joule heating and (**b**) after Joule heating XPS C 1s spectra of CNT films (**c**) before Joule heating and (**d**) after Joule heating.

To further investigate the structural and chemical evolution during Joule heating, we performed TEM and XPS characterization on the samples before and after the treatment. HRTEM images (Figure 5a) reveal that the pristine CNT sample contains noticeable amorphous carbon deposits on the tube surfaces. After Joule heating (Figure 5b), these amorphous carbon layers are largely removed. Furthermore, the diameters of the carbon nanotubes showed no significant change after Joule heating. The inner diameter is approximately 5 nm. The majority of the tubes are double-walled carbon nanotubes, with some being multi-walled, and the outer diameter ranges from approximately 5 to 20 nm. XPS C 1s spectra (Figure 5c,d) were deconvoluted into graphitic sp^2^ carbon (~284.5 eV), sp^3^-hybridized carbon (~285.4 eV), C=O (~286.5 eV), and Fe-C (~283.8 eV) components. The sp^2^/sp^3^ ratio increases from 6.09 in the pristine sample to 7.71 after Joule heating, indicating a net increase in graphitic sp^2^ domains. Concurrently, the C=O component, which is associated with oxygen-containing defects, decreases from 7.6% to 3.3%, suggesting the effective removal of defective carbon species during Joule heating. These observations are consistent with the Raman results (narrowed G-band, increased I_G_/I_D_ and I_2D_/I_G_ ratios). Combining the TEM, XPS and Raman spectra results, we suggest that Joule heating promotes stronger inter-tube interactions, likely through the removal of amorphous carbon barriers and defective carbon species, thereby facilitating more intimate and ordered contacts between adjacent tubes. This interfacial enhancement, together with impurity removal and tube-wall defect repair, contributes to efficient load transfer and suppression of inter-tube sliding during tensile deformation, as reflected in the improved mechanical performance.

At 0.5 A (≈1800 °C), the decrease in mechanical properties (Figure S3c) is attributed to excessive thermal energy, which can cause localized carbon vaporization, the formation of new vacancies or etch pits on the tube walls, and over-graphitization that makes the network brittle. Therefore, the optimal Joule heating condition (0.3 A, ≈1000 °C) strikes a balance between activating atomic diffusion and avoiding thermal damage.

The microstructural changes induced by Joule heating were examined via SEM and Raman spectroscopy. Before Joule heating, the CNT strips exhibited well-aligned but physically contacted tubes with clear inter-tube boundaries (Figure 4e). After Joule heating at the optimal condition (0.3 A, ≈1000 °C), the tube junctions became less distinct, and continuous carbon networks formed at the contact points (Figure 4f). This morphology indicates that carbon atoms at the tube junctions gained sufficient thermal energy to migrate and re-organize, creating bridged structures that enhanced inter-tube load transfer.

Raman spectroscopy (Figure 4g) provided further evidence of structural repair. The I_G_/I_D_ ratio increased from 2.33 (for the sample after acid washing and pre-stretching) to 3.12 after Joule heating, indicating a significant reduction in lattice defects (vacancies, dangling bonds and topological defects). Detailed analysis of the Raman spectra reveals that the full width at half maximum (FWHM) of the G-peak decreased significantly from approximately 50 cm^−1^ in the pristine sample to approximately 26 cm^−1^ after Joule heating, while the I_2D_/I_G_ ratio increased from approximately 0.38 to 0.48, further confirming the restoration of graphitic ordering and the conjugated carbon structure. The G-peak position showed a slight downshift, suggesting enhanced π-π interactions and improved graphitic ordering. These changes confirm that Joule heating effectively repaired tube-wall defects and promoted graphitization.

**Figure 6:**
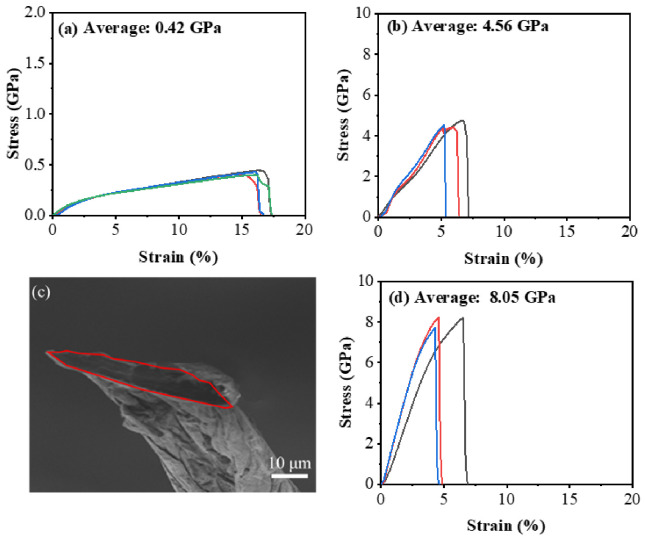
True tensile strengths of different treatments. (**a**) Pristine film (0.42 GPa); (**b**) After Joule heating (4.56 GPa); (**c**) Cross-section after rolling; (**d**) After rolling (8.05 GPa).

To obtain the true tensile strength, the mechanical properties were evaluated via tensile testing and cross-sectional SEM observation. As shown in Figure 6a, the pristine CNT film exhibited a tensile strength of 0.42 GPa. After the three-step treatment (acid washing, pre-stretching, and Joule heating), the tensile strength significantly increased to 4.56 GPa (Figure 6b). Additional rolling further densified the film. The cross-sectional SEM image (Figure 6c) reveals a compact structure, and the corresponding tensile stress-strain curve (Figure 6d) gives a tensile strength of 8.05 GPa. These results demonstrate that the imposed post-treatments dramatically enhanced the mechanical performance of CNT films through purification, defect repair, interfacial enhancement, and densification.

**Figure 7:**
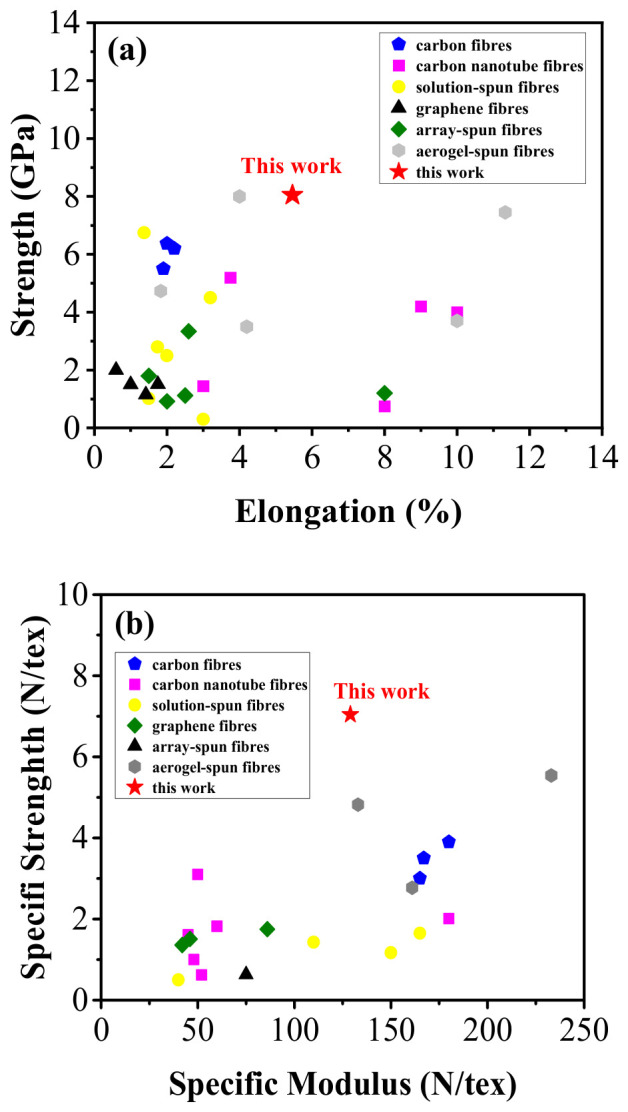
Comparison of mechanical performance of CNT strips from this work with representative previous carbon materials. (**a**) Tensile strength vs. elongation at break. (**b**) Specific strength vs. specific modulus. Blue pentagons: carbon fibers; grey hexagons: carbon nanotube fibers; yellow circles: solution-spun fibers; black triangles: graphene fibers; green diamonds: array-spun fibers; magenta squares: aerogel-spun fibers; red star: this work. All data are from references [21,22,23,24,25,26,27,28,29,30,31,32,33,34,35,36,37,38].

To benchmark the mechanical performance achieved in this work, Figure 7 compares the tensile strength, elongation, specific strength, and specific modulus of our CNT strips with previous representative results for carbon fibers, graphene fibers, and other CNT fibers. Consistent with the literature used for comparison, we also employed conventional tensile testing to failure to obtain the mechanical properties, with a strain rate of 0.01 s^−1^ and a typical strip geometry for the samples. Furthermore, to avoid discrepancies in the property results arising from different calculation methods, we adopted the approach most commonly used in the literature, i.e., calculating the strength as the maximum force divided by the cross-sectional area. Meanwhile, to eliminate the influence of density on the evaluation of mechanical properties, we also compared the specific strength and specific modulus of our materials with those of relevant materials reported in the literature in Figure 7b. In Figure 7a, our material (red star) exhibits a tensile strength of 8.05 GPa with an elongation at break of approximately 5.46%. In contrast to most high-strength carbon fibers (which typically exhibit elongations of <2%), our CNT strips achieve a combination of high strength and enhanced deformability, avoiding the extreme brittleness commonly seen in commercial carbon fibers. In Figure 7b, our CNT strips show a specific strength of 7.04 N/tex and a specific modulus of 129 N/tex. These values are significantly higher than most solution-spun, array-spun, and aerogel-spun CNT fibers, and are comparable to or even exceed those of some commercial carbon fibers. It should be noted that the literature data cited in Figure 7 originate from different laboratories with varying density calculation approaches (e.g., linear density vs. cross-sectional area-based methods), which may introduce deviations in the absolute values of specific strength and specific modulus. Nevertheless, the performance enhancement achieved through our three-step post-treatment strategy (from 0.64 N/tex for the pristine sample to 7.04 N/tex) substantially exceeds the typical variation caused by these methodological differences, confirming that the improvement in mechanical properties is significant and reliable. Furthermore, the tensile strength of 8.05 GPa obtained in this work already surpasses that of some commercial carbon fibers cited in Figure 7 (e.g., T300-grade, ~3.5 GPa) and most previously reported CNT fibers (typically in the range of 1–5 GPa), further demonstrating the practical potential of this strategy for producing high-performance CNT macrostructures.

### 3.4. Mechanistic Analysis

**Figure 8:**
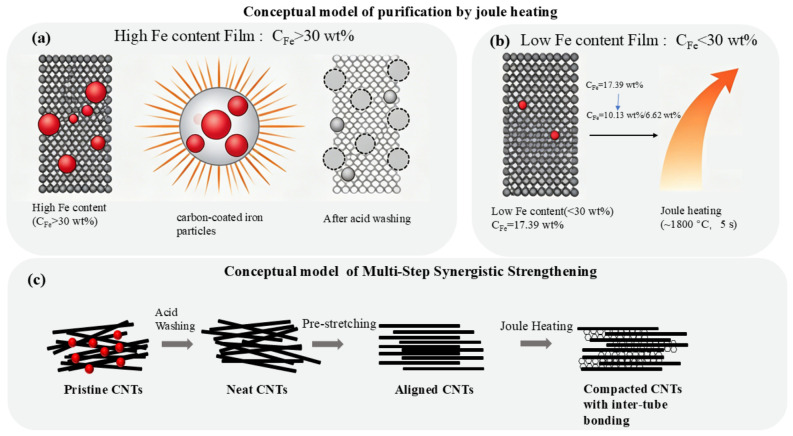
Conceptual model illustrating the proposed strengthening mechanisms based on experimental observations. (**a**) Purification conceptual model for high-iron films during direct Joule heating; (**b**) purification conceptual model for low-iron films during direct Joule heating; (**c**) synergistic strengthening conceptual model of the three-step post-treatment process (acid washing, pre-stretching, and Joule heating). It should be noted that the proposed mechanisms are inferred from the experimental evidence presented in this work and are intended as a conceptual framework rather than fully demonstrated phenomena.

The mechanical enhancement achieved in this study arises from four synergistic effects: impurity removal, defect repair, interfacial enhancement, and densification. These mechanisms are summarized schematically in Figure 8 and are discussed below with reference to the experimental data.

Joule heating removes iron impurities through two distinct pathways depending on the initial iron content. For high-iron films (Fe > 30 wt.%), the temperature exceeds the melting point of iron. The molten iron migrates to the CNT surface and solidifies into spherical particles [39,40]. During this process, amorphous carbon dissolves in the molten iron and precipitates as graphitic shells, forming a Fe/Fe_3_C@C core–shell structure (Figure 2e). Subsequent acid washing selectively dissolves the iron core, leaving hollow carbon shells attached to the CNT network (Figure 2h). For low-iron films (Fe < 30 wt.%), no visible particles form; instead, the iron content decreases from 17.39 wt.% to 6.62 wt.% (Figure 2i), indicating removal via atomic diffusion and evaporation at the higher temperature (≈1800 °C) [41,42]. In both cases, elimination of iron impurities removes stress concentration sites that would otherwise weaken the mechanical performance. However, experimental results indicated that the high temperature which removal of iron impurities required could cause unavoidable damage to the CNTs, resulting in a marked decline in mechanical performance that could not be reversed by any subsequent processing. Consequently, we opted for Joule heating at a relatively lower temperature (0.3 A, 1000 °C) to improve the mechanical properties and employed acid washing for purification instead.

In addition to impurity removal, Joule heating repairs atomic-scale defects in the CNT walls. Raman spectroscopy (Figure 4g) shows that the I_G_/I_D_ ratio increases from 2.33 (after acid washing and pre-stretching) to 3.12 after Joule heating, indicating healing of vacancies, dangling bonds and topological defects [16,43,44]. The narrowing of the G-peak full width at half maximum and the slight downshift of the G-peak further suggest improved graphitic ordering and stronger π-π interactions. Defect repair restores the intrinsic load-bearing capacity of individual CNTs [45,46,47].

A critical effect of Joule heating is the enhancement of inter-tube interfaces. At tube junctions, the local electrical resistance is higher than that of the tube bodies, generating stronger Joule heating and creating microscopic hot spots. At ≈1000 °C, carbon atoms at these contact points migrate across the original physical interface and reorganize into continuous carbon networks. Before Joule heating, the aligned CNTs show clear inter-tube gaps (Figure 4e); after heating, these boundaries become indistinct and the network appears fused (Figure 4f). This morphological change suggests that weak van der Waals contacts are replaced by stronger, likely covalent-like bonds [48]. Consequently, load transfer efficiency improves and inter-tube sliding during tensile deformation is suppressed, as reflected in the increased tensile load (from 289.82 cN to 322.23 cN) and the decreased elongation at break (from 7.29% to 5.70% at the optimum condition).

Pre-stretching aligns the CNTs along the stretching direction, as shown by SEM and polarized Raman spectroscopy (Figure 4a–d). However, the aligned tubes are still only in physical contact. Joule heating induces thermal stress that radially compresses the network, reducing the linear density from 0.75 tex (after pre-stretching) to 0.42 tex (after Joule heating). Moreover, the strong inter-tube bonds formed during heating lock the oriented structure, preventing the CNTs from relaxing back to a disordered state after unloading. This locking effect explains why the specific strength increases from 5.57 N/tex (pre-stretched only) to 7.04 N/tex (after Joule heating).

The three steps are synergistic rather than additive. Acid washing alone removes impurities but leaves the CNTs disordered and weakly interfaced, yielding a specific strength of 2.96 N/tex. Pre-stretching alone aligns the tubes but does not heal defects or create strong bonds, reaching 5.57 N/tex. Joule heating alone (without pre-cleaning and alignment) is less effective because impurities hinder direct tube-tube contacts and random orientation limits the number of load-bearing junctions. Only the combination of all three steps produces the outstanding mechanical properties: a specific strength of 7.04 N/tex and a true tensile strength of 4.56 GPa (further increased to 8.05 GPa by optional rolling, Figure 6d).

When the Joule heating current exceeds the optimum (0.5 A, ≈1800 °C), the mechanical properties degrade (Figure S3). Excessive thermal energy possibly causes localized carbon vaporization, creating new vacancies and etch pits on the tube walls, and may also lead to over-graphitization that makes the network brittle. However, direct experimental evidence for this degradation mechanism is not yet available and further investigation is needed. Therefore, a moderate temperature (≈1000 °C) is essential to achieve beneficial effects without inducing structural damage.

In summary, the three-step strategy provides a fast and controllable route to high-performance CNT films. By sequentially addressing impurities, alignment, defects and interfaces, this multi-scale engineering elevates the mechanical properties of macroscopic CNT assemblies far beyond those of the pristine material.

## 4. Conclusions

In this work, we have developed a three-step synergistic strategy of acid washing, pre-stretching and Joule heating to significantly enhance the mechanical properties of CNT films prepared via FCCVD. The main conclusions are as follows. Direct Joule heating can effectively purify CNT films, and the purification mechanism depends on the iron content. For high-iron films (Fe > 30 wt.%), iron melts, migrates to the surface and forms Fe/Fe_3_C@C core–shell particles, which can be converted into hollow carbon shells via acid washing. For low-iron films (Fe < 30 wt.%), iron is removed via atomic diffusion and evaporation without forming visible particles; TGA confirms that the iron content decreases from 17.39 wt.% to 6.62 wt.%. However, the high temperature (1400 °C) required for purification inevitably caused a marked decrease in mechanical properties, which could not be reversed. Therefore, acid washing with 30 vol.% CSA, which removes impurities without severely damaging the CNT network, becomes a favorable alternative, increasing the specific strength from 0.64 N/tex to 2.96 N/tex. Pre-stretching induces alignment of the CNTs along the stretching direction, further raising the specific strength to 5.57 N/tex. The optimal Joule heating condition (0.3 A, ≈1000 °C, 3 s) repairs lattice defects (I_G_/I_D_ increases from 2.33 to 3.12), transforms the weak van der Waals contacts between tubes into continuous carbon networks, densifies the network (linear density decreases from 0.75 tex to 0.42 tex) and locks the aligned structure. The specific strength reaches 7.04 N/tex and the true tensile strength reaches 4.56 GPa (8.05 GPa after rolling). Overheating (0.5 A, ≈1800 °C) leads to property degradation, which may be attributed to carbon vaporization and new defect formation. The three steps are synergistic rather than additive; only the combination of impurity removal, alignment, defect repair, interfacial enhancement, densification and orientation locking yields the outstanding mechanical performance.

This work demonstrates that a fast, controllable Joule heating treatment, in combination with simple chemical and mechanical pre-treatments, can transform a weak, impure and disordered CNT assembly into a high-performance structural material. The proposed strategy is expected to be applicable to other carbon nanomaterial assemblies.

## Data Availability

The original contributions presented in this study are included in the article/Supplementary Material. Further inquiries can be directed to the corresponding author.

## Supplementary Materials

The following supporting information can be downloaded at: https://www.mdpi.com/article/10.3390/ma19132917/s1.
