# Supplementary material for: Large Improvement of the Mechanical Strength of Carbon Nanotube Films by Joule Heating Dominated Post Treatments
Source: Materials (Basel). 2026 Jul 7;19(13):2917. doi: 10.3390/ma19132917 (PMC13362599; doi:10.3390/ma19132917)
Supplement: Supplementary file 1 [file materials-19-02917-s001.zip › materials-4414838-supplementary.pdf]

# Supplementary Materials

# Large improvement of the mechanical strength of carbon nanotube films by Joule heating dominated post treatments

**Zujia Hu[1], Yifan Feng[1], Heng Zhang[1], Kangfei Liu[2], Xinran Cheng[1], Yunxiao Du[1] and Jiannong Wang[2,*]**

[1] School of Materials Science and Engineering, East China University of Science and Technology, 130 Meilong Road, Shanghai, 200237, China
[2] School of Mechanical and Power Engineering, East China University of Science and Technology, 130 Meilong Road, Shanghai, 200237, China
* Corresponding author. E-mail address: jnwang@ecust.edu.cn (J.N. Wang).

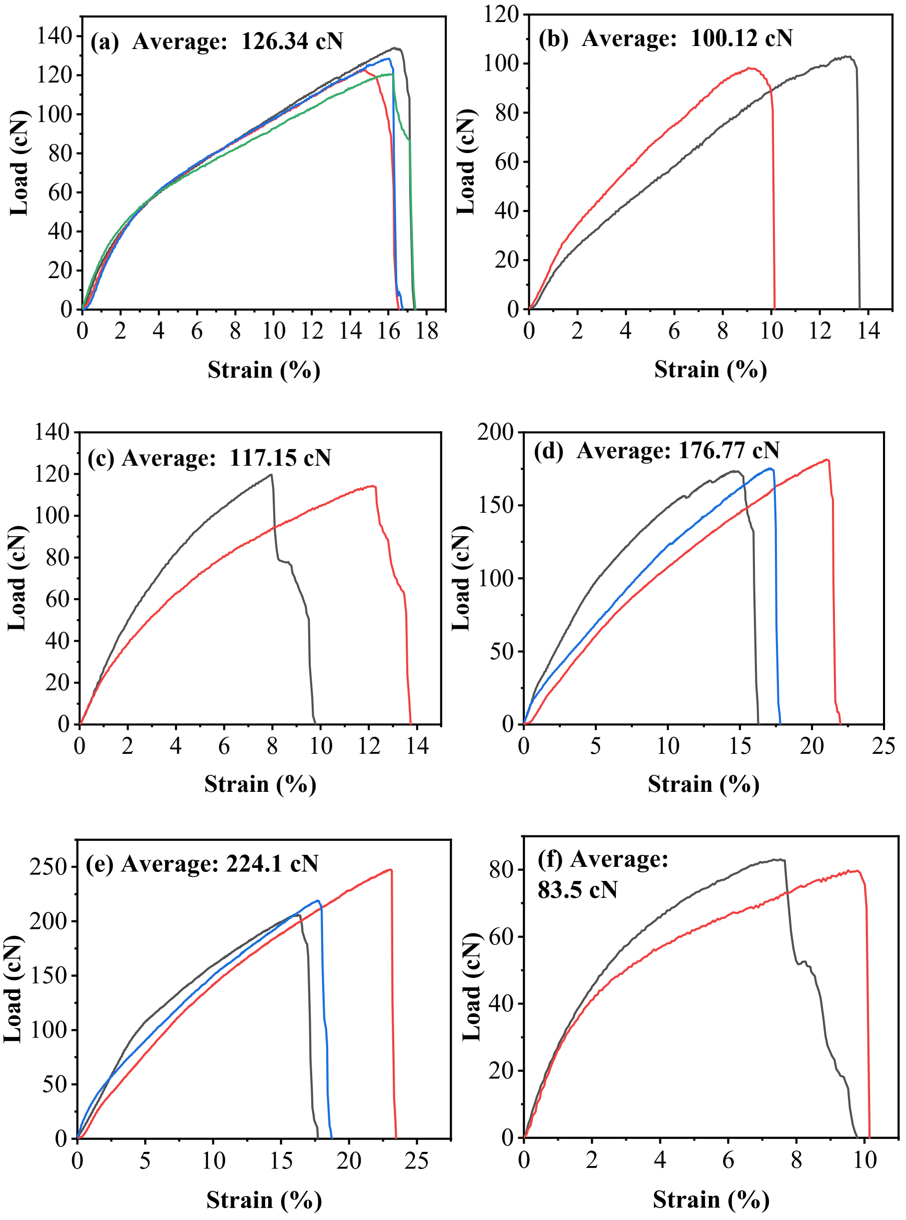


**Figure S1.** Tensile load-strain curves of CNT strips after acid washing with different CSA concentrations (additional data). Curves are shown for (a) 0 vol%, (b) 5 vol%, (c) 10 vol%, (d) 20 vol%, (e) 30 vol%, and (f) 50 vol% CSA.

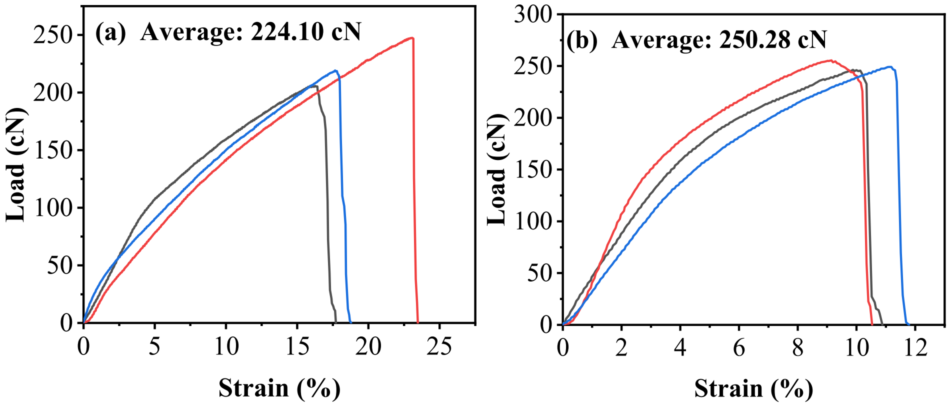

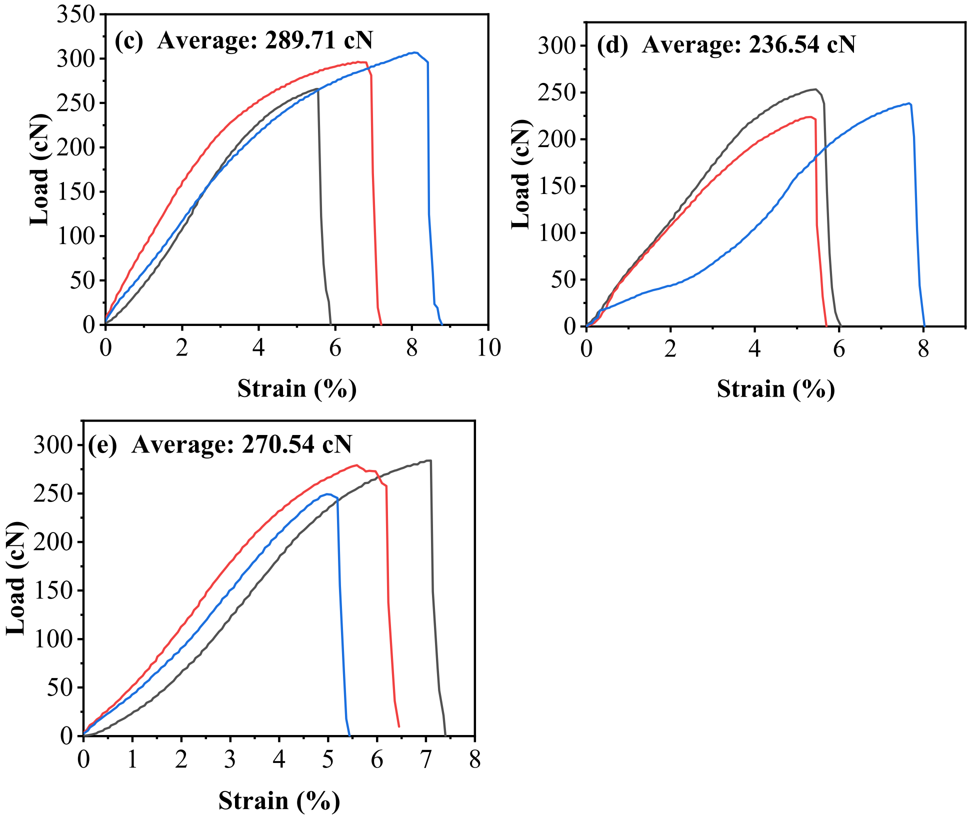


**Figure S2.** Tensile load-strain curves of CNT strips after pre-stretching with different loads (additional data). Data for loads of (a) 0, (b) 50 g, (c) 100 g, (d) 150 g and (e)100 g for 3 h are presented.

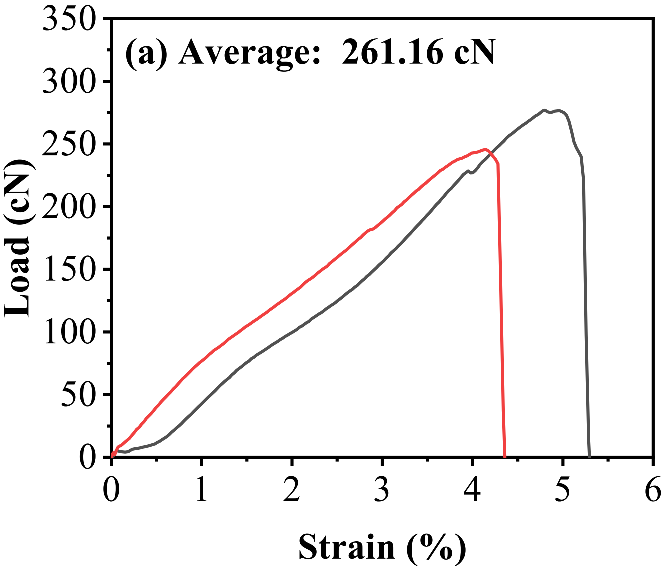

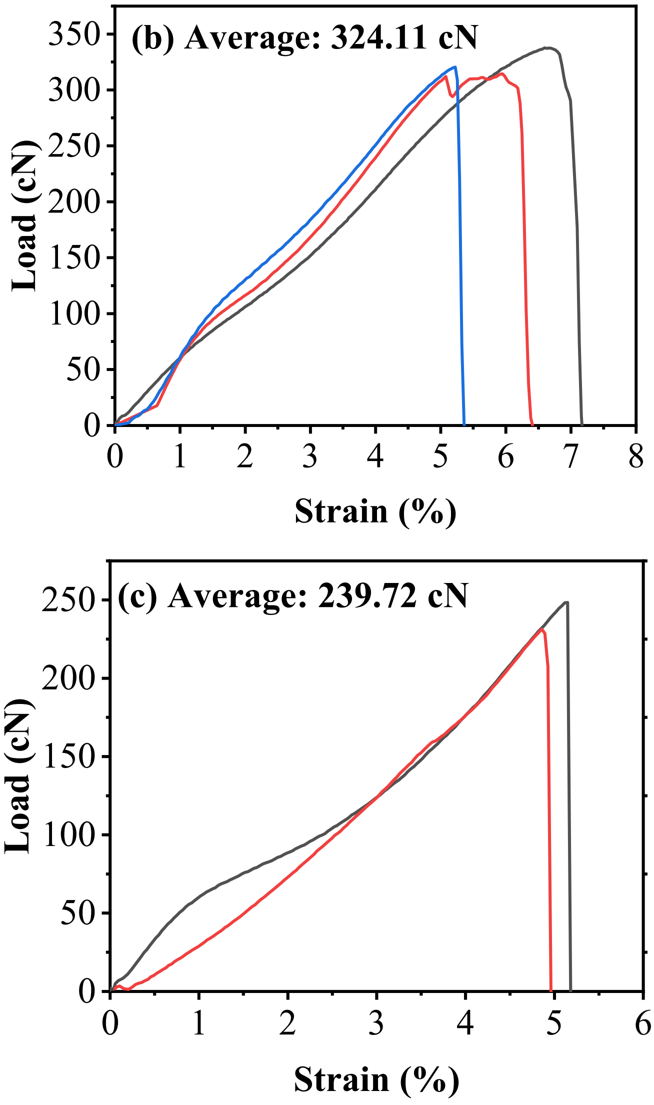


**Figure S3.** Tensile load-strain curves of CNT strips after Joule heating at different currents (additional data). (a) Curves for 0.1 A (≈800 °C), (b) 0.3 A (≈1000 °C), and (c) 0.5 A (≈1800 °C).
